# Lithium Diffusion in Silicon Encapsulated with Graphene

**DOI:** 10.3390/nano11123397

**Published:** 2021-12-15

**Authors:** Wei Qin, Wen-Cai Lu, Xu-Yan Xue, Kai-Ming Ho, Cai-Zhuang Wang

**Affiliations:** 1 College of Physics, Qingdao University, Qingdao 266071, China; xuexy@qdu.edu.cn; 2 State Key Laboratory of Bio-Fibers and Eco-Textiles, Qingdao University, Qingdao 266071, China; 3 Institute of Theoretical Chemistry, Jilin University, Changchun 130021, China; 4 Ames Laboratory-U.S. DOE and Department of Physics and Astronomy, Iowa State University, Ames, IA 50011, USA; kmh@iastate.edu

**Keywords:** perfect graphene (*p*–Gr), defective graphene (*d*–Gr), Gr/Si slab, diffusion barrier, CI-NEB calculation

## Abstract

The model of a graphene (Gr) sheet putting on a silicon (Si) substrate is used to simulate the structures of Si microparticles wrapped up in a graphene cage, which may be the anode of lithium-ion batteries (LIBS) to improve the high-volume expansion of Si anode materials. The common low-energy defective graphene (*d*–Gr) structures of DV5–8–5, DV555–777 and SV are studied and compared with perfect graphene (*p*–Gr). First-principles calculations are performed to confirm the stable structures before and after Li penetrating through the Gr sheet or graphene/Si-substrate (Gr/Si) slab. The climbing image nudged elastic band (CI-NEB) method is performed to evaluate the diffusion barrier and seek the saddle point. The calculation results reveal that the *d*–Gr greatly reduces the energy barriers for Li diffusion in Gr or Gr/Si. The energy stability, structural configuration, bond length between the atoms and layer distances of these structures are also discussed in detail.

## 1. Introduction

Facing the ever-growing demands for electrical vehicles and portable electronic devices [1,2,3], rechargeable lithium-ion batteries (LIBS) with high energy density, long cycle life and fast charge rate have become the focus of intense research. Silicon is an attractive and promising anode material for LIBS due to its high theoretical capacity (~4200 mAh/g) [4], over ten times higher than conventional graphite, and abundance on Earth. However, the large-volume expansion of silicon as an anode material upon lithiation (~300%) [5,6] in practical applications leads to fracture and loss of inter-particle electrical contact, consequently causing early capacity fading, thus blocking the further improvement of LIBS. Attempting to avoid the mechanical fracture via decreasing the material size, the use of nanostructure silicon (nano–Si) has been shown to be fruitful, such as in silicon crystalline amorphous core-shell nanowires [7], interconnected Si hollow nanosphere [8], Si nanotubes [9], and so on. In particular, Si–C nanostructure materials combining Si with carbonaceous materials [10,11,12,13,14,15,16,17,18,19,20,21,22,23,24] can retain capacity well, further enhancing the cycling stability and charging rate [10,25] due to the buffering effect and high electrical conductivity of carbon. On the other hand, a large number of theoretical calculations have been focused on the interactions between Li and Si crystals or between Li and graphite separately [26,27,28,29,30,31,32,33,34], or on the influence of Si–C composites on lithiation [35]. Despite the impressive improvement of the stability of Si anodes after adopting nano-Si, the Coulombic efficiency is still far lower than conventional graphite due to the large surface area. In addition, the complex synthesis process makes nano-Si costly. These are the major obstacles to the further development and mass production of LIBS technology. Subsequently, Si microparticles were explored to replace nano-Si as an anode material due to the lower cost in manufacture, but the cycling performance is far poorer than that of nano-Si. After the first few cycles, Si microparticles are pulverized into smaller sizes, turn into amorphous particles from Si crystalline, and then lose electrical contact [36,37,38,39].

In recent years, a strategy of Si microparticles surrounded by a porous carbon matrix was used to counteract these inadequacies. The combination maintains both Li absorption capacity and electrical conductivity well for many battery-charging cycles. For example, a conformal growth of the conductive graphene cage, which can wrap up Si microparticles, was reported as a promising encapsulation material [40]. The similar surface chemistry to graphite allows graphene cage to form a stable solid electrolyte interphase [41,42]. Being mechanically strong and flexible, graphene cages remain undamaged with Si microparticles fracturing and confine all the fractured Si pieces within the cages, thus maintaining the essential electrical contact between broken Si particles within [40]. Graphene-encapsulated Si microparticles thus provide a new architecture of materials for the development of LIBS technology. A theoretical understanding of Li diffusion in such materials could therefore provide information and guidance for the design and development of LIBS, but the relevant calculated studies are scarce. Odbadrakh et al. reported that cavities along the reconstructed Si surface provide diffusion paths for Li [43]. Chou and Hwang investigated the role of the interface in lithiation of silicon/graphene (Si/Gr) [44] composites and demonstrated the charge transfer from Li to both silicon and graphene. They also showed that Li cations exhibit substantially higher mobility along the Si/Gr interface than that of bulk Si [44]. For Li diffusion, most studies focused on diffusion paths of Li on the graphene surface [45,46,47]. However, the difficulty of Li intercalating through the graphene sheet to bind to Si particles, i.e., the diffusion barrier during lithiation, is deeply influenced by the surface characteries of graphene for such a combination. It is well known that many defects will inevitably occur in the process of graphene producing. Can these defects be used to facilitate the diffusion of Li? If so, which common defects are easier for Li to intercalate through to reduce the diffusion barrier of Li? Do Si particles affect the diffusion? Does the diffusion barrier increase or decrease in the presence of Si particles? Studying these issues could assist in exploring more suitable encapsulation materials.

Usually, single–vacancy (SV), Stone–Wales (SW) and divacancy (DV) defects are typical point defects in graphene and the subjects of intense research [48,49,50,51,52,53,54]. SV and SW defects are created by removing a C atom and rotating a C–C bond by 90° in the plane, respectively. The DV defect is formed by removing a C–C dimer; when the dimer is removed from graphene, a series of reconstructions around the DV defect take place upon annealing. In this work, the Gr/Si (a monolayer graphene is put on the silicon substrate) is used to simulate the Si microparticles encapsulated with the graphene cage. The Si substrate and defective graphene (*d*–Gr) are adopted to simulate Si particles and porous graphene, respectively. The perfect graphene (*p*–Gr) is also studied as a comparison. The energy stability of these models (both Gr and Gr/Si, Gr = *p*–Gr, DV5–8–5, DV555–777 and SV) are investigated by first-principles calculations. Their energy barriers of Li diffusion through them are also determined.

## 2. Computational Methods

**Figure 1:**
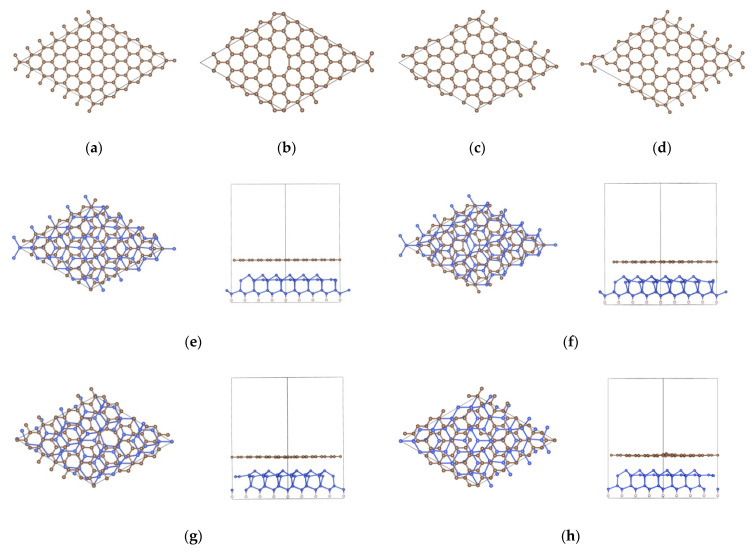
Structures of (**a**) *p*–Gr, (**b**) DV5–8–5, (**c**) DV555–777, (**d**) SV, (**e**) *p*–Gr/Si, (**f**) DV5–8–5/Si, (**g**) DV555–777/Si and (**h**) SV/Si; the left and right configurations (**e**–**h**) are the top and side views of Gr/Si, respectively.

**Figure 2:**
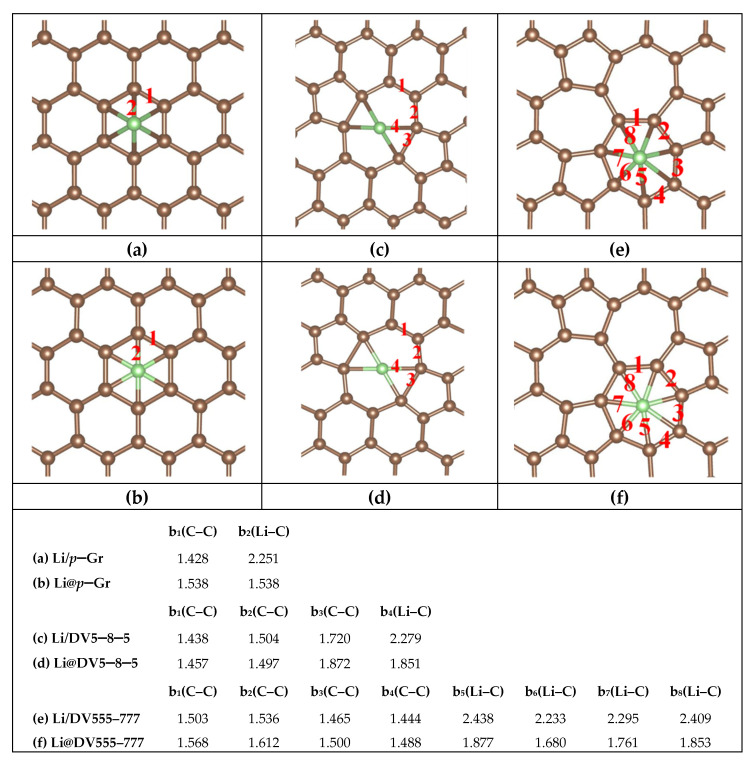
Structures of (**a**) Li/*p*–Gr (**b**) Li@*p*–Gr (**c**) Li/DV5–8–5 (**d**) Li@DV5–8–5 (**e**) Li/DV555–777 and (**f**) Li@DV555–777; their main bond lengths (abbreviated “b” in the list, Å) are shown in the list at the bottom of the figure. The red numbers in these figures correspond to the subscripts in the list.

**Figure 3:**
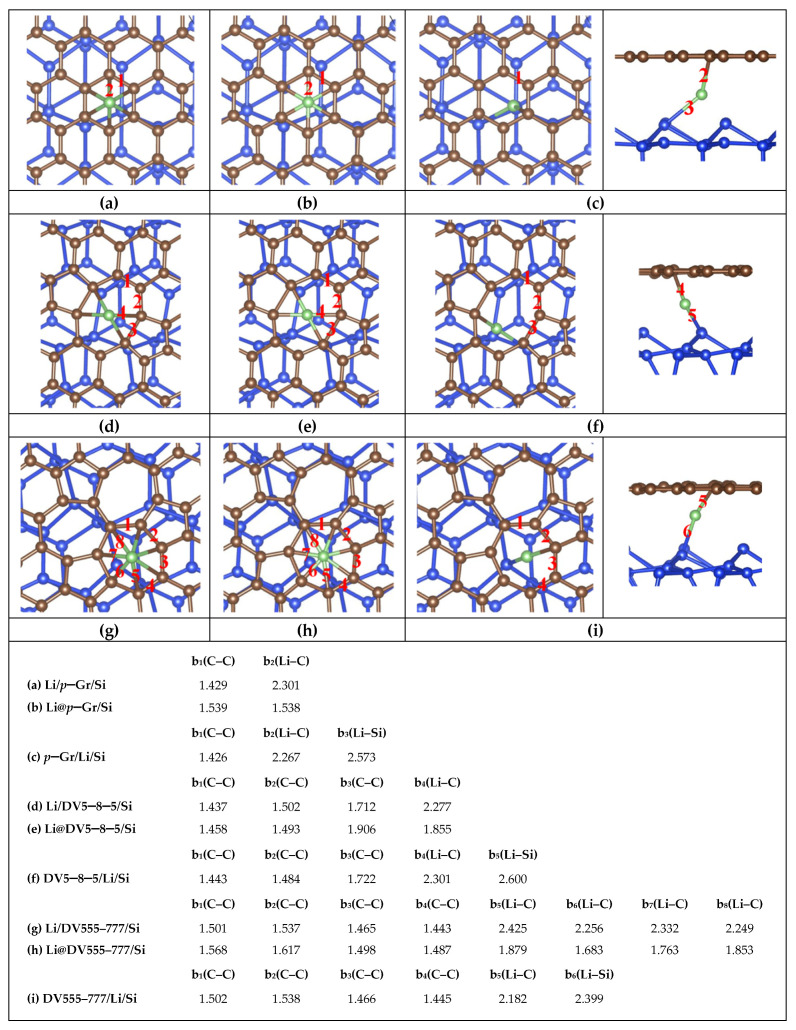
Structures of (**a**) Li/*p*–Gr/Si (**b**) Li@*p*–Gr/Si (**c**) *p*–Gr/Li/Si (**d**) Li/DV5–8–5/Si (**e**) Li@DV5–8–5/Si (**f**) DV5–8–5/Li/Si (**g**) Li/DV555–777/Si (**h**) Li@DV555–777/Si and (**i**) DV555–777/Li/Si. The left and right configurations (**c**,**f**,**i**) are the top and side views of the end structures in the CI-NEB calculation, respectively. The main bond lengths (abbreviated as “b” in the list, Å) are shown in the list at the bottom of the figure. The red numbers in these figures correspond to the subscripts.

**Figure 4:**
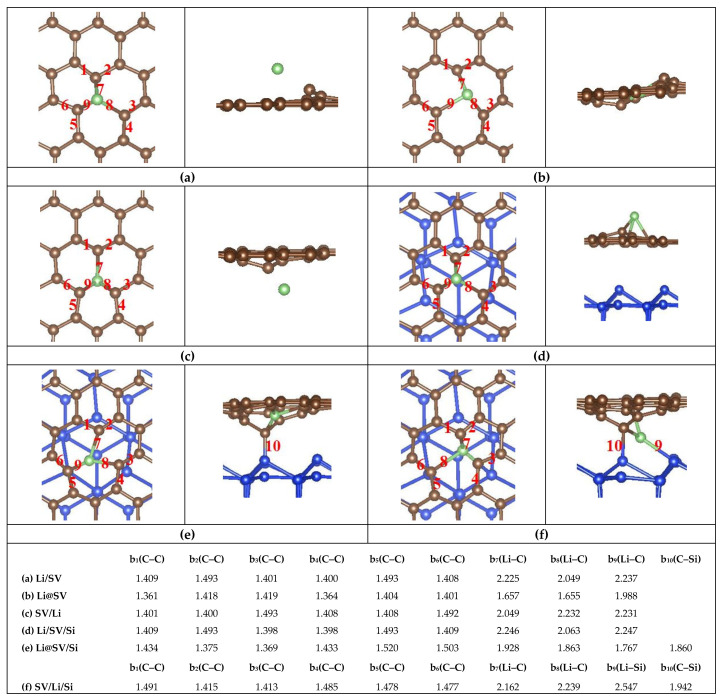
Structures of (**a**) Li/SV (**b**) Li@SV (**c**) SV/Li (**d**) Li/SV/Si (**e**) Li@SV/Si and (**f**) SV/Li/Si. The left and right configurations are the top and side views of the six structures in the CI-NEB calculation, respectively. The main bond lengths (abbreviated as “b” in the list, Å) are shown in the list at the bottom of the figure. The red numbers in these figures correspond to the subscripts.

Considering both the calculation cost and accuracy, a 6 × 6 hexagonal supercell of *d*–Gr is employed to model the porous graphene cage. Three common defects [55], including the SV defect, DV5–8–5 defect (containing two pentagons and one octagon) and DV555–777 defect (three heptagons in the center surrounded by three pentagons) are considered. A 4 × 4 supercell of the Si (111) surface containing two double atomic layers are employed as the Si substrate. The silicon dangling bonds on the bottom are passivated by hydrogen atoms, and these hydrogen atoms and silicon atoms on the bottom are fixed. The Si–Si bond lengths in Si substrate are adjusted by about 4% to fit the 6 × 6 Gr sheet to minimize the effect of the lattice mismatch on graphene when the in-plane periodic boundary conditions are used. To eliminate the interaction between Gr (or Gr/Si) and their periodic replicas, the vacuum layers of 20 Å and 18 Å are adopted in the perpendicular direction to the Gr surface for monolayers Gr and Gr/Si, respectively. The structures of Gr and Gr/Si are displayed in Figure 1. All calculations are performed using the projector-augmented wave (PAW) with Perdew, Burke, and Ernzerh of (PBE) GGA functional for the exchange-correlation energy [56,57], as implemented in the Vienna ab initio simulation package (VASP) [58]. We also consider the Van der Waal’s force followed by the well-established Grimme’s DFT-D2 formula [59,60] in the VASP calculation for all the Gr/Si. The Brillouin zone (BZ) integration is approximated by using the Gamma centered k-grid. For structural relaxation and energy evaluation, the 8 × 8 × 1 k-grid and 4 × 4 × 1 k-grid are adopted for the monolayer Gr and Gr/Si slab, respectively. We also calculate the energy barriers of Li diffusion in Gr and Gr/Si, and the climbing image nudged elastic band (CI-NEB) method implemented in the VASP code is used to seek the saddle points and the minimum energy path. As a reference, we study the adsorption sites about Li on the surfaces of perfect graphene (*p*–Gr) and *p*–Gr/Si. Three typical adsorption sites are considered: the top (T) site (Li is above a C atom), the bridge (B) site (Li is above the midpoint of a C–C bond) and the hollow (H) site (Li is above the center of a hexagon ring). The results show that the H site is the most stable adsorption site. Similarly, in *d*–Gr and *d*–Gr/Si (Gr = DV5–8–5, DV555–777 and SV), the structures are more stable when Li is above the center of the polygon rings. These lowest-energy structures are shown in Figure 2, Figure 3 and Figure 4 as the initial configurations of the CI-NEB calculations. We call them Li/Gr and Li/Gr/Si (Gr = *p*–Gr, DV5–8–5, DV555–777 and SV), respectively. When Li penetrates through the Gr sheet, Li is basically on the symmetric sites of the other surface of the graphene due to the planar structures of the monolayer Gr sheet. Their configurations are similar to the initial points from the top view, while for Gr/Si, Li is between the Gr sheet and Si substrate after passing through the Gr layer. We consider various relative sites between Li and Si atoms, such as Li above a Si atom, Li above a Si–Si bond, Li above a folded Si hexagon ring, and so on. All of the candidates are relaxed to find the lowest-energy structures. These lowest-energy structures after Li intercalates into Gr or Gr/Si are adopted as the end points and called Gr/Li and Gr/Li/Si (Gr = *p*–Gr, DV5–8–5, DV555–777 and SV), respectively. In the CI-NEB calculation, four images are employed in addition to the initial and end points. The structures of the saddle points are found and called Li@Gr and Li@Gr/Si (Gr = *p*–Gr, DV5–8–5, DV555–777 and SV), respectively. They are also displayed in Figure 2, Figure 3 and Figure 4.

In order to evaluate the energetic stability of different *d*–Gr and *d*–Gr/Si (Gr = DV5–8–5, DV555–777 and SV), we firstly calculate their formation energies (*E*_f_). For monolayer Gr sheets, their *E*_f_ is defined as

*E*_f_ = *E*_d_ − *N μ*_C_ (1)

and for *d*–Gr/Si slab, the *E*_f_ is defined as

*E*_f_ = *E*_d_ − *E*_sub_ − *N μ*_C_ (2)

where *E_d_* and *E*_sub_ are the total energy of *d*–Gr (or *d*–Gr/Si) and Si substrate in the given supercells, respectively; *N* is the number of C atoms in the supercell, and *μ*_C_ is the chemical potential of carbon, which is leveled from perfect graphene with the same unit cell size. Our calculation results showed that the formation energies (*E*_f_) of DV5–8–5, DV555–777 and SV are 7.645, 6.386 and 7.579 eV, respectively, which is in agreement with previous studies [51,59]. The formation energies (*E*_f_) of DV5–8–5/Si, DV555–777/Si and SV/Si are 2.621, 1.254 and 1.906 eV, respectively. The order of *E*_f_ does not alter with the existence of the Si substrate.

To examine the stability of the structures upon Li adsorption, we also calculate the adsorption energies (*E*_ads_) of Li/Gr and Li/Gr/Si (Gr = *p*–Gr, DV5–8–5, DV555–777 and SV) which are defined as

*E*_ads_ = *E*_t_ (Li–S) − *E*_t_ (S) − *E*_Li_ (3)

where *E*_t_ (Li–S) and *E*_t_ (S) are the total energies of Gr (or Gr/Si) with and without a Li atom adsorbing on their surfaces, respectively. *E*_Li_ is the energy of putting one Li atom into the same unit cell size. The results of the Li adsorption energies (*E*_ads_) are listed in the second column of Table 1. The lower the *E*_ads_, the more stable the adsorption structure (Li/Gr and Li/Gr/Si). From Table 1, we can see that for monolayer Gr (Gr = *p*–Gr, DV5–8–5, DV555–777 and SV), Li prefers to adsorb on the *d*–Gr (Gr = DV5–8–5, DV555–777 and SV), compared with *p*–Gr. The *E*_ads_ of *d*–Gr is more than 1 eV lower than that of *p*–Gr. In particular, Li/SV is the most stable adsorption structure among the four sheets, whose *E*_ads_ is nearly 1.8 eV lower than that of *p*–Gr. For Li/Gr/Si, the order of their *E*_ads_ is consistent with that of Li/Gr, except Li/SV/Si. Comparing with *p*–Gr/Si, Li prefers to adsorb on DV5–8–5/Si and DV555–777/Si. The differences of their *E*_ads_ are not as large as that between monolayer *p*–Gr and *d*–Gr, about 0.5 eV. It indicates that the differences of *E*_ads_ are reduced between *p*–Gr/Si and *d*–Gr/Si (*d*–Gr = DV5–8–5 and DV555–777) due to the Si substrate. However, it is difficult to adsorb on SV/Si for Li. The *E*_ads_ of Li/SV/Si is even higher, about 1 eV, than that of Li/*p*–Gr/Si. It may be related to the structural deformation of SV/Si when Li adsorbs on it (detailed in Results and Discussion).

The formation energies (*E*_f_) of Gr/Li/Si (Gr = *p*–Gr, DV5–8–5, DV555–777 and SV) are also calculated when Li intercalates the Gr sheet and is located between Gr and the Si substrate, which is defined as

*E*_f_ = *E*_t_ (Gr/Li/Si) − *E*_t_ (Gr) − *E*_t_ (Si) − *E*_Li_ (4)

where *E*_t_ (Gr/Li/Si) is the total energies of the Gr/Li/Si (Gr = *p*–Gr, DV5–8–5, DV555–777 and SV) intercalated structures; *E*_t_ (Gr) and *E*_t_ (Si) are the total energies of the Gr sheet and Si substrate, respectively. In order to measure the energy stability of the structures before and after Li intercalation, we also calculate their energy differences (*E*_diff_), which are defined as

*E*_diff_ = *E*_f_ (Li/Gr/Si) − *E*_f_ (Gr/Li/Si) (5)

where *E*_f_ (Li/Gr/Si) and *E*_f_ (Gr/Li/Si) are the formation energies of Li/Gr/Si and Gr/Li/Si (Gr = *p*–Gr, DV5–8–5, DV555–777 and SV) calculated in the same method (Formula (4)). The values of *E*_f_ of Gr/Li/Si and *E*_diff_ are shown in the third and fourth columns of Table 1, respectively. The results show that the structures after Li passes through the Gr sheet are more stable than those of the adsorption on the Gr surface. It may be because Li combines with both C atoms in the Gr and Si atoms on the surface of the Si substrate when Li passes through the Gr sheet, which make the structure more stable. The difference value of *p*–Gr/Si is the largest. It indicates that the stability of the intercalated structure (*p*–Gr/Li/Si) after Li passes through *p*–Gr is much higher than that of the adsorption on the Gr surface (Li/*p*–Gr/Si). By comparison, the *E*_diff_ value for *d*–Gr/Si is relatively low.

## 3. Results and Discussion

### 3.1. Energetic Stability

Figure 2, Figure 3 and Figure 4 show the most stable structures of Li adsorbed on different Gr and Gr/Si (Gr = *p*–Gr, DV5–8–5, DV555–777 and SV) surfaces. From the figures, we can see that Li prefers to absorb above the center of the polygon ring with the most sides so that it can have more carbon neighbors. For instance, the adsorption energies (*E*_ads_) are lower when Li absorbs above the hexagon ring center on the *p*–Gr and *p*–Gr/Si surfaces. Similarly, the lowest-energy adsorption sites are above the centers of the heptagon and octagon rings for DV555–777 (or DV555–777/Si) and DV5–8–5 (or DV5–8–5/Si) structures, respectively. For SV (or SV/Si), the most stable adsorption site is just above the C atom, removed from the SV defect. When Li is diffused into the Gr (or Gr/Si), Li is in the plane of Gr for the saddle-point structures. After penetrating through the Gr sheet, Li is basically on the symmetric sites of the other surface of the Gr sheet, and in Gr/Si, the positions of Li are slightly shifted from the top view. The Gr (Gr = *p*–Gr, DV5–8–5 and DV555–777) layer maintains a planar structure throughout, whether there is the Si substrate or not (see Figure 2 and Figure 3). The SV defect is a little special. When Li is adsorbed on the surface of SV, the C atoms in the SV are no longer in the same plane. One of the C atoms is obviously deviated from the SV plane and approaches the Li atom, whether in Li/SV or Li/SV/Si. After Li penetrates through SV, the SV layer is also non-planar, whether for SV/Li or SV/Li/Si. In SV/Li, one C atom is close to the Li atom; more C atoms deviate from the SV plane and approach the Li atom and Si surface in SV/Li/Si. For the saddle-point structures, the Li atom is basically in the initial plane of SV, but the C atoms around Li are almost deviated from the original plane. In Li@SV, these C atoms are distributed on both sides of the plane, while in Li@SV/Si, they are all close to the side of the Si surface (see Figure 4).

In the bottom of Figure 2, Figure 3 and Figure 4, the relevant bond lengths, including b(C–C), b(Li–C) and b(Li–Si) are also listed. The C–C bond length (b_1_(C–C) in Figure 2a) of the hexagon ring absorbed by Li in Li/*p*–Gr is 1.428 Å. The corresponding value is 1.429 Å (b_1_(C–C) in Figure 3a) when there is the Si substrate, which is basically unchanged. Similar behavior is seen in DV5–8–5 and DV555–777 (see Figure 2 and Figure 3). We also find the similar regular pattern in their saddle-point structures Li@Gr/Si (Gr = *p*–Gr, DV5–8–5 and DV555–777). The maximum variation of the C–C bond length is about 0.03 Å when there is the Si substrate. It indicates that the Si substrate has negligible effects on the C–C bond lengths. Furthermore, the C–C bond lengths in the saddle-point structures are larger than the ones of the corresponding initial structures Li/Gr or Li/Gr/Si (Gr = *p*–Gr, DV5–8–5 and DV555–777). When Li penetration is complete, the C–C bond lengths are basically restored to the values of the initial structures. It may be due to Li being in the plane of Gr in the saddle-point structures, while Li leaves the plane of Gr in Gr/Li or Gr/Li/Si (Gr = *p*–Gr, DV5–8–5 and DV555–777). Similarly, the Li–C bond lengths also vary very little when there is the Si substrate. Among the three Gr sheets (Gr = *p*–Gr, DV5–8–5 and DV555–777), the Li–C bond lengths’ value of DV555–777 changes most. Despite all this, its average Li–C bond length changes less than 0.03 Å when there is the Si substrate, compared with the values b_5–8_(Li–C) in Li/DV555–777 (from 2.233 Å to 2.438 Å in Figure 2e) and Li/DV555–777/Si (from 2.249 Å to 2.425 Å in Figure 3g). Comparing with the three Gr (or Gr/Si) above, there are some different results due to the C atoms around Li deviating from the SV plane when Li diffuses in the SV (or SV/Si). The average bond lengths of C–C and Li–C in the saddle-point structure Li@SV do not increase although the Li atom is in the SV plane but decrease. After Li penetrates the SV plane, both C–C and Li–C bond lengths completely return to the initial values. For SV/Si, the results are slightly complicated due to the existence of Si substrate. The average bond length of C–C in the saddle-point structure Li@SV/Si increases slightly (~0.006 Å) compared to the value of the initial structure (Li/SV/Si), while the average bond length of Li–C decreases over 0.3 Å compared to the initial structure. After Li penetrates the SV plane, the positions of the C atoms around Li change greatly, and the average bond length of C–C does not return to the initial value, but is increased by about 0.03 Å. Similar to the other three Gr/Si, both the C and Si atoms bond with the Li atom at this time. In addition, the shortest bond length of Li–C in Li/SV (b_8_(Li–C) in Figure 4a) is 2.049 Å, which is lower than the values in other Li/Gr (b_2_(Li–C) in Figure 2a, b_4_(Li–C) in Figure 2c and b_6_(Li–C) in Figure 2e). The closer combination between Li and C in Li/SV can reduce *E*_ads_. The order of *E*_ads_ does not change when putting these Gr sheets on the Si substrate, except SV/Si. It is the most difficult to adsorb on the surface of SV/Si for the Li atom in these four slabs, which may be due to the structural deformation of SV/Si when Li adsorbs on it.

As a reference, we also calculate the layer distances of each structure, displayed in Table 2. We first calculate the average coordinates of all the C atoms in the Gr (Gr = *p*–Gr, DV5–8–5, DV555–777 and SV) sheet and Si atoms on the surface of the Si substrate as the positions of the Gr sheet and Si surface, respectively. Then the distances between the Li atoms, Gr sheet and Si surface are calculated and labeled as *d*_C–Si_, *d*_Li–C_ and *d*_Li–Si_ in Table 2. The distances between the Gr sheet and the surface of Si substrate (*d*_C–Si_) in most Gr/Si slabs change little upon Li adsorption. Especially for DV555–777/Si, its *d*_C–Si_ value is almost unchanged before and after Li adsorption. However, the *d*_C–Si_ value of SV/Si decrease by about 0.1 Å upon Li adsorption, which is about 10 times that of other structural variation. It indicates that the adsorption of Li has the greatest effect on the bonding between SV and the surface of the Si substrate among the four Gr above, while the effect is the smallest in DV555–777/Si. In Gr/Li/Si (Gr = *p*–Gr, DV5–8–5, DV555–777 and SV), Li is located between the Gr and the surface of the Si substrate as Li diffuses into the Gr. Although there is one more Li atom between them, compared with Gr/Si, most *d*_C–Si_ values of Gr/Li/Si do not increase but decrease, except Li/DV555–777/Si. In sum, the adsorption of Li has a slight effect on the *d*_C–Si_ values in *p*–Gr/Si and DV5–8–5/Si, and the effect disappears after Li diffuses into *p*–Gr/Si (or DV5–8–5/Si). For DV555–777/Si, when Li is adsorbed on the DV555–777 surface, the *d*_C–Si_ value is almost unchanged. However, when Li diffuses into DV555–777, the distance between DV555–777 and the surface of Si substrate is widened. Nevertheless, its *d*_C–Si_ value is still the smallest of the corresponding four slabs, whether in Gr/Si, Li/Gr/Si, or Gr/Li/Si (Gr = *p*–Gr, DV5–8–5, DV555–777 and SV). In these Gr/Si slabs, the adsorption of Li has the greatest influence on the *d*_C–Si_ value of SV/Si. The *d*_C–Si_ value decreases when Li is adsorbed on the SV/Si surface; its value further decreases when Li passes through the SV. The total reduction is more than 0.2 Å. It may be due to the larger change of the C positions in SV during Li diffusion to SV/Si. The existence of the Si substrate also slightly affects the distances (*d*_Li–C_) from the Li to Gr sheet. With the Si substrate, the values of *d*_Li–C_ increase for Li/*p*–Gr/Si but decrease for Li/*d*–Gr/Si (Gr = DV5–8–5, DV555–777 and SV). The value of *d*_Li–C_ in Li/SV/Si reduces most, about 0.1 Å. For monolayer Gr (Gr = *p*–Gr, DV5–8–5, DV555–777 and SV), the *d*_Li–C_ values are basically unchanged due to Li being in the symmetrical position of Li/Gr after Li passes through the Gr sheet. Nevertheless, the *d*_Li–C_ values in Gr/Li/Si increase, except DV555–777/Li/Si, when there is the Si substrate. The *d*_Li–C_ value in DV5–8–5/Li/Si increase the most, over 0.4 Å. Considering the distance between Gr and the surface of the Si substrate (*d*_C–Si_), Li is generally closer to the Si surface, except DV555–777/Li/Si. In proportion, the distance from Li to Gr is SV > *p*–Gr > DV5–8–5 > DV555–777. Comparing with the other three Gr/Si slabs, Li in DV555–777/Li/Si is closer to DV555–777. From this point of view, Li seems to prefer to bond to DV555–777, or it may be less easily bonded to the Si substrate in DV555–777/Si. For *d*_Li–Si_ in Gr/Li/Si (Gr = *p*–Gr, DV5–8–5, DV555–777 and SV), in proportion, the order is the opposite of *d*_Li–C_ mentioned above, i.e., SV < *p*–Gr < DV5–8–5 < DV555–777. The layer distances indirectly show the bonding between Li and Gr (or Si substrate) in the four Gr/Si slabs above, which can help us better understand the energy stability of these structures.

From the bond lengths and layer distances analysis above, we can see that the SV defect is special. Although the structural stability of Li/SV is high, the adsorption and diffusion of Li have a great impact on the planar structure of SV when the Si substrate exists. In the process of Li diffusion, the position of the C atom around the defect, structural configuration, bond lengths and layer distances in SV/Si changes greatly. In other words, the structure of SV/Si changes greatly, and its structural stability is low during Li diffusion in it. For the other three Gr/Si slabs, the structural configurations, bond lengths and layer distances are relatively stable during Li diffusion in them, that is, the influence of the Si substrate on them can be basically ignored.

### 3.2. Li Diffusion in Gr and Gr/Si (Gr = p–Gr, DV5–8–5, DV555–777 and SV)

**Figure 5:**
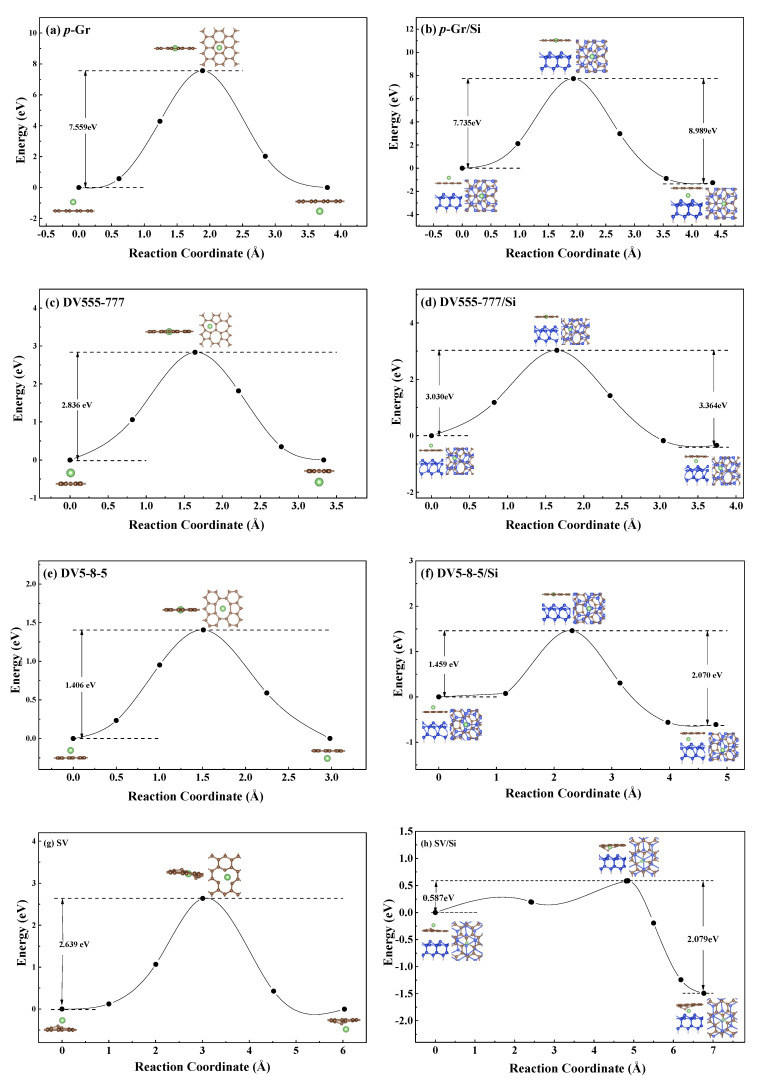
Energy barrier of Li diffusion.

In order to better illustrate the difficulty of Li diffusion in Gr and Gr/Si (Gr = *p*–Gr, DV5–8–5, DV555–777 and SV), we calculate their diffusion barrier as shown in Figure 5. As a reference, we firstly study Li diffusion in *p*–Gr and *p*–Gr/Si. It is the most stable structure when Li is adsorbed on the H site of *p*–Gr and the *d*_Li–C_ is about 1.755 Å as shown in Table 2, which is chosen as the initial point (Li/*p*–Gr) in the CI-NEB calculations. The end point (*p*–Gr/Li) is the symmetric adsorption position on the other side of the *p*–Gr plane. The calculation results show that the energy barrier of the Li diffusion in *p*–Gr is as high as 7.559 eV, shown in Figure 5a, which indicates that it is quite difficult for Li to penetrate through the *p*–Gr sheet. For *p*–Gr/Si, the most stable structure when Li is adsorbed on it (Li/*p*–Gr/Si shown in Figure 3a) is still chosen as the initial point, and *p*–Gr/Li/Si in Figure 3c is the end point in the CI-NEB calculation after Li intercalates *p*–Gr. From Figure 5, we can see that the energy barrier for Li diffusion in *p*–Gr/Si is higher (0.176 eV) than in the case of the *p*–Gr sheet. That is, the presence of the Si substrate increases the energy barrier for Li penetration through *p*–Gr. These calculation results suggest that in the model (Si microparticles encapsulated with graphene cage), it is very difficult for Li to diffuse into the *p*–Gr layer when Si particles are encapsulated with *p*–Gr. Furthermore, it is even more difficult for Li to diffuse out once it is intercalated between *p*–Gr and the Si substrate since the energy barrier of diffusion is higher, shown in Figure 5b. It can be seen that it does not seem to be a good choice to encapsulate Si particles by *p*–Gr. In that way, what about the common defective graphene (*d*–Gr)?

Defects will inevitably occur in the process of graphene producing, and DV5–8–5, DV555–777 and SV are the common low-energy defect structures on graphene. Therefore, we also study the diffusion behavior of Li diffusion in *d*–Gr and *d*–Gr/Si (Gr = DV5–8–5, DV555–777 and SV), which is shown in Figure 5c–h. In the CI-NEB calculations, the lowest-energy structures of Li adsorbed on *d*–Gr (or *d*–Gr/Si) are used as the initial point structures. Similar to *p*–Gr, for *d*–Gr, their end points are the symmetric adsorption position on the other side of the plane, and for *d*–Gr/Si, they are the *d*–Gr/Li/Si structures shown in Figure 3 and Figure 4. The calculation results show that the defective graphenes greatly reduce the energy barriers when Li diffusion in graphene. Comparing with the energy barrier of Li diffusion in *p*–Gr, the corresponding values of DV555–777 and SV are significantly lower by ~4.723 eV and ~4.92 eV, respectively. The value of DV5–8–5 decreases the most, and its diffusion barrier is only 1.406 eV, which is lower (~6.153 eV) than the value of *p*–Gr. How about encapsulating Si particles using these defective graphenes since defects can reduce the energy barrier of Li diffusion into graphene? *d*–Gr/Si is used to simulate the encapsulating structures. When Li diffuses in DV555-777/Si and DV5–8–5/Si, the energy barriers are slightly increased compared with the case of monolayer DV555-777 and DV5–8–5. Nevertheless, their diffusion barriers decrease by 4.705 eV and 6.276 eV than the value in *p*–Gr/Si, respectively. For SV/Si, the energy barrier of Li diffusion to it is unexpectedly decreased to 0.587 eV. It is probably because the position of the C atom around the SV defect changes greatly during Li diffusion, and SV no longer maintains planar configuration. In addition, the structures of Gr/Li/Si are more stable after Li penetrating in the Gr sheet than adsorption on the surface of Gr/Si, whether for *p*–Gr/Si or *d*–Gr/Si. It indicated that it is more difficult for Li to diffuse out from Gr/Si. However, it is much easier for Li to diffuse out from *d*–Gr/Si than from *p*–Gr/Si, shown in Figure 5. From the perspective of the diffusion barrier, the SV defective graphene seems to be a good material for encapsulating Si particles. However, our calculation results suggest that the C atoms and surface Si atoms around SV defects in SV/Si greatly shift during Li diffusion. The times of charge and discharge are reduced and the battery life is shortened if Si particles are encapsulated with SV. On the other hand, the energy barrier of Li diffusion from SV/Si is not low; it is even slightly higher than the value of DV5–8–5/Si. In brief, the probability of Li penetration through graphene would be greatly enhanced if Si particles are encapsulated with defective graphene instead of perfect graphene. Considering the energy barriers of Li diffusion into and out from Gr/Si and the stability of the structures of Gr/Si during Li diffusion, DV5–8–5 defective graphene may be a good choice as the material for encapsulated Si particles.

In order to better understanding the electronic structures of DV5–8–5, we also calculate the Bader charge, band structures and PDOS of these structures during Li diffusion in DV5–8–5 and DV5–8–5/Si. As a reference, the electronic structures of corresponding *p*–Gr are also calculated, which are shown in the supplementary material. The DV5–8–5 defect exhibits a Dirac-like cone band structure, but a gap opens between the Dirac-like cones as shown in Figure S2a. There is a nearly flat band above the E_F_ level and within the gap between the Dirac-like cones. When the Li atom adsorbs on it (i.e., Li/DV5–8–5), the band in the gap becomes more dispersive and partially occupied as shown in Figure S2b. Meanwhile, the gap between the Dirac-like cones reduces. With the diffusion of the Li atom (Li@DV5–8–5), the gap between the Dirac-like cones becomes wider as shown in Figure S2c. After Li penetration is completed, the band structure is recovered as shown in Figure S2b. During Li diffusion, the Li atom maintains the Li^+^ ion. When the Si substrate is included, the DV5–8–5 still exhibits a Dirac-like cone structure. The gap between the cones becomes wider. There are three flat bands in the gap due to the influence of the Si substrate, and these bands are all above E_F_ as shown in Figure S2d. During Li diffusion in DV5–8–5/Si, the system remains as p–type doping; the Li atom maintains the Li^+^ ion; the bands in the gap become more complex; and some of the bands become more dispersive and partially occupied. Similar to the case of DV5–8–5, the gap between the Dirac-like cones experiences a process of first reducing (Li/DV5–8–5/Si), then increasing (Li@DV5–8–5/Si), and finally basically recovering (DV5–8–5/Li/Si).

## 4. Conclusions

During Li diffusion in the Gr sheet and Gr/Si slab (Gr = *p*–Gr, DV5–8–5, DV555–777 and SV), the initial points, saddle points and end structures are studied by first-principles calculation. Firstly, the initial and end points of different structures are screened, and their energetic stability is also discussed. Then the CI-NEB calculations are employed to evaluate the diffusion barrier by seeking the saddle point and the minimum energy path. For monolayer Gr, Li prefers to adsorb on *d*–Gr (Gr = DV5–8–5, DV555–777 and SV), comparing with *p*–Gr. The *E*_ads_ of Li/DV5–8–5 and Li/DV555–777 is more than 1 eV lower than that of Li/*p*–Gr; the *E*_ads_ of the most stable adsorption structure Li/SV is nearly 1.8 eV lower than that of Li/*p*–Gr. The *E*_ads_ order of Li/Gr/Si is consistent with that of Li/Gr, except Li/SV/Si. It is difficult to adsorb on SV/Si for Li, as its *E*_ads_ is even higher about 1 eV than that of Li/*p*–Gr/Si. It may be related to the structural deformation of SV/Si. During Li diffusion in Gr, the Gr sheets basically maintain the planar whether for Gr or Gr/Si, except for SV. When Li diffuses in SV, one of the C atoms is obviously deviated from the SV plane and approaches the Li atom whether in the initial point Li/SV or the end point SV/Li. More C atoms deviate from the SV plane and approach the Li atom when Li diffuses in SV/Si. Even in the saddle point Li@SV and Li@SV/Si, the C atoms around Li are almost deviated from the original plane, i.e., during Li diffusion in SV and SV/Si, the SV plane structure is seriously deformed; moreover, several Si atoms on the surface of Si substrate deviate from their original positions in SV/Li/Si. It may be the reason why many properties of Li diffusing in SV or SV/Si are different from those of the other Gr, including the order of *E*_ads_, bond lengths between atoms, layer distance and even the diffusion barrier. For monolayer Gr (Gr = *p*–Gr, DV5–8–5 and DV555–777), the average bond lengths of C–C and Li–C in the saddle-point structures (Li@Gr) are larger than the ones of the corresponding initial structures (Li/Gr) due to Li being in the plane of Gr. When Li penetration is complete, the bond lengths are basically restored to the values of the initial structures. In Gr/Si, the Si substrate has negligible effects on these bond lengths. Comparing with the three defects above, the average C–C and Li–C bond lengths of the saddle-point structure Li@SV are not increased but decreased, although the Li atom is also in the SV plane. In Li@SV/Si, the average C–C bond length increases slightly, while its Li–C average bond length decreases over 0.3 Å compared to the initial structure. After Li penetrates the SV plane, the C–C average bond length does not return to the initial value but is increased. The distances between the Gr sheet and the surface of the Si substrate (*d*_C–Si_) in Gr/Si (Gr = *p*–Gr, DV5–8–5 and DV555–777) change little during Li diffusion in them. However, the *d*_C–Si_ value of SV/Si decreases about 0.1 Å upon Li adsorption, which is about 10 times that of other structural variation. When Li passes through the SV, its *d*_C–Si_ value further decreases, and the total reduction is more than 0.2 Å. The existence of the Si substrate also slightly affects the distances (*d*_Li–C_) from Li to the Gr sheet. Comparing with Li/Gr (Gr = *p*–Gr, DV5–8–5, DV555–777 and SV), the *d*_Li–C_ in Li/*p*–Gr/Si increases, while decreasing in Li/*d*–Gr/Si. The *d*_Li–C_ in Li/SV/Si decreases most, about 0.1 Å. After passing through Gr, Li is generally closer to the Si surface, except for DV555–777/Li/Si. Considering the distance between Gr and the Si surface (*d*_C–Si_), the distance from Li to Gr is SV > *p*–Gr > DV5–8–5 > DV555–777 in proportion. Accordingly, the order of *d*_Li–Si_ is opposite to that above. It indirectly provides evidence that Li binds more closely to the Si surface in SV/Li/Si while bonding more tightly to the DV555–777 layer in DV555–777/Li/Si. The CI-NEB calculation results show that *d*–Gr greatly reduces the energy barriers whether Li diffuses into Gr or Gr/Si. The energy barrier of Li diffusion in DV5–8–5 is just 1.406 eV, which is lower by ~6.153 eV than the value of *p*–Gr. When Li diffuses into Gr/Si, the energy barriers are slightly increased comparing with the case of monolayer Gr. Nevertheless, the diffusion barriers of *d*–Gr/Si are still much lower than the value in *p*–Gr/Si. The energy barrier of Li diffusion to SV/Si is unexpectedly decreased to 0.587 eV. In addition, the structures of Gr/Li/Si are more stable than Li/Gr/Si whether for *p*–Gr or *d*–Gr, which indicates that it is more difficult for Li to diffuse out from Gr/Si. However, it is much easier for Li to diffuse out from *d*–Gr/Si than from *p*–Gr/Si. Our results suggest that the Si particles encapsulated by defective graphene may be a candidate for anode materials of LIBS. From the perspective of the diffusion barrier, both SV and DV5–8–5 defective graphenes seem to be good materials for encapsulated Si particles, and SV is better. However, the SV/Si structure is deformed greatly during Li diffusion, which reduces the times of charge and discharge and shortens the battery life. Moreover, the energy barrier of Li diffusion out from SV/Si is not low and is even slightly higher than the value of DV5–8–5/Si. In contrast, DV5–8–5/Si remains a relatively stable structure during Li diffusion within it. Therefore, DV5–8–5 defective graphene may be a good choice as the material of encapsulated Si particles.

## Figures and Tables

**Table 1:** Adsorption energies (*E*_ads_, eV) of the initial structures and the formation energies (*E*_f_, eV) of the end structures with Li diffusion in Gr and Gr/Si (Gr = *p*–Gr, DV5–8–5, DV555–777 and SV); the energy differences (*E*_diff_, eV) between the initial and end structures with Li diffusion in Gr/Si (Gr = *p*–Gr, DV5–8–5, DV555–777 and SV).

Structures	*E*_ads_ (eV)	*E*_f_ (eV)	*E*_diff_ (eV)
Li/*p*-Gr	−1.308		
Li/DV5–8–5	−2.340		
Li/DV555–777	−2.505		
Li/SV	−3.065		
Li/*p*–Gr/Si	−4.135		
Li/DV5–8–5/Si	−4.552		
Li/DV555–777/Si	−4.693		
Li/SV/Si	−3.154		
*p*–Gr/Li/Si		−9.322	1.254
DV5–8–5/Li/Si		−10.189	0.610
DV555–777/Li/Si		−10.170	0.334
SV/Li/Si		−9.601	0.374

**Table 2:** The layer distances (Å) of Gr and Si surface of Si substrate (*d*_C–Si_) and the distances (Å) between Li atom and Gr (*d*_Li–C_) or Si surface (*d*_Li–Si_) during Li diffusion in Gr and Gr/Si (Gr = *p*–Gr, DV5–8–5, DV555–777 and SV).

Structures	Distances (Å)		
	*d*_C–Si_	*d*_Li–C_	*d*_Li–Si_
*p*–Gr/Si	3.567		
DV5–8–5/Si	3.400		
DV555–777/Si	3.233		
SV/Si	3.555		
Li/*p*–Gr		1.755	
Li/DV5–8–5		1.427	
Li/DV555–777		1.565	
Li/SV		1.930	
Li/*p*–Gr/Si	3.577	1.778	
Li/DV5–8–5/Si	3.384	1.416	
Li/DV555–777/Si	3.236	1.546	
Li/SV/Si	3.426	1.833	
*p*–Gr/Li/Si	3.565	2.045	1.520
DV5–8–5/Li/Si	3.397	1.862	1.534
DV555–777/Li/Si	3.267	1.471	1.796
SV/Li/Si	3.337	2.014	1.323

## Data Availability

Not applicable.

## Supplementary Materials

The following are available online at https://www.mdpi.com/article/10.3390/nano11123397/s1: Table S1: The bader charges of Li atom, Gr (Gr = *p*–Gr and DV5–8–5) sheet and Si substrate of the initial, saddle and end structures during Li diffusion in Gr and Gr/Si. Figure S1: Band structures of (a) *p*–Gr (b) Li/*p*–Gr (c) Li@*p*–Gr (d) Si Substrate (e) *p*–Gr/Si (f) Li/*p*–Gr/Si (g) Li@*p*–Gr/Si (h) *p*–Gr/Li/Si. Figure S2: Band structures of (a) DV5–8–5 (b) Li/DV5–8–5 (c) Li@DV5–8–5 (d) DV5–8–5/Si (e) Li/DV5–8–5/Si (f) Li@DV5–8–5/Si and (g) Li/DV5–8–5/Si. Figure S3: The line configurations of the initial, saddle and end structures during Li diffusion in Gr and Gr/Si (Gr = *p*–Gr and DV5–8–5); and the typical C atoms discussed in Figures S4–S11 are labeled. The green ball represents Li atom; and the brown and blue lines represent C atoms and Si atoms and their bonding, respectively. Figure S4: PDOS of the typical C atoms (labeled in Figure S3) of p–Gr and all Si atoms of Si substrate. Figure S5: PDOS of Li atom and the typical C atoms (labeled in Figure S3) in (a) Li/p–Gr and (b) Li@p–Gr. Figure S6: PDOS of the typical C atoms (labeled in Figure S3) and all Si atoms in p–Gr/Si. Figure S7: PDOS of Li atom, all Si atoms and the typical C atoms (labeled in Figure S3) in (a) Li/p–Gr/Si (b) Li@p–Gr/Si and (c) p–Gr/Li/Si. Figure S8: PDOS of the typical C atoms in DV5–8–5 labeled in Figure S3. Figure S9: PDOS of Li atom and the typical C atoms (labeled in Figure S3) in (a) Li/DV5–8–5 and (b) Li@DV5–8–5. Figure S10: PDOS of all Si atoms of the typical C atoms (labeled in Figure S3) in DV5–8–5/Si. Figure S11: PDOS of Li atom, all Si atoms and the typical C atoms (labeled in Figure S3) in (a) Li/DV5–8–5/Si (b) Li@DV5–8–5/Si and (c) DV5–8–5/Li/Si.
