# Supplementary material for: Lithium Diffusion in Silicon Encapsulated with Graphene
Source: Nanomaterials (Basel). 2021 Dec 15;11(12):3397. doi: 10.3390/nano11123397 (PMC8709124; doi:10.3390/nano11123397)
Supplement: Supplementary file 1 [file nanomaterials-11-03397-s001.zip › nanomaterials-1483834-supplementary.pdf]

nanomaterials

Supplementary Materials

Lithium Diffusion in Silicon Encapsulated with Graphene

Wei Qin [1,2,*], Wen-Cai Lu [1,2,3,*], Xu-Yan Xue [1,2], Kai-Ming Ho [4] and Cai-Zhuang Wang [4,*]

[1] College of Physics, Qingdao University, Qingdao 266071, China; xuexy@qdu.edu.cn
[2] State Key Laboratory of Bio-Fibers and Eco-Textiles, Qingdao University, Qingdao 266071, China
[3] Institute of Theoretical Chemistry, Jilin University, Changchun 130021, China
[4] Ames Laboratory-U.S. DOE and Department of Physics and Astronomy, Iowa State University, Ames, IA 50011, USA; kmh@iastate.edu
* Correspondence: qinw@qdu.edu.cn (W.Q.); wencailu@jlu.edu.cn (W.-C.L.); wangcz@ameslab.gov (C.-Z.W.)

In this supporting information, we provide the bader charges of Li atom, Gr sheet and Si substrate of the initial, saddle and end structures during Li diffusion in Gr and Gr/Si (Gr = *p*−Gr and DV5−8−5) in Table S1. Their band structures are showed in Figures S1 − S2. Figures S3 − S11 mark the typical C atoms of these structures and show their PDOS, respectively.

It is well known that the Fermi level (E_F) of the *p*−Gr is in accordance with the Dirac point as shown in Figure S1a. When Li atom adsorbed on the H site of *p*−Gr (Li/*p*−Gr), almost all its valence electron on the 2s state transfers to the π* bands of *p*−Gr, and shifts the E_F of the *p*−Gr above the Dirac point by ~ 0.31 eV as shown in Table S1 and Figure S1b. Meanwhile a tiny gap (~ 0.02 eV) opens at the Dirac point. The gap of the saddle point structure (Li@*p*-Gr) becomes wider (~ 0.13 eV) and the system remain n-type doping as shown in Figure S1c. When Li penetration is completed, the band structure is the same as that of the initial configuration as shown in Figure S1b due to the equivalence of the adsorption sites on the two side of the *p*−Gr sheet. When Si substrate is included, the *p*−Gr still exhibits the Dirac cone structure but the E_F is slightly below the Dirac point by ~ 0.09 eV (i.e., p-doped) as shown in Figure S1e. There is also a tiny gap (0.08 eV) at the Dirac point. Upon Li adsorption, the E_F shifts above the Dirac point by about 0.19 eV (i.e., n-doped) as shown in Figure S1f, which is smaller than the corresponding value in the case without Si substrate (Li/*p*−Gr). This is because the valence electron from Li atom transfers almost equally to both *p*−Gr and Si substrate as one can see the list in Table S1. Similar to the case of Li@*p*−Gr, a band gap (~ 0.13 eV) of Li@*p*−Gr/Si also opens and the *p*−Gr is substantially n-doped with the E_F laid ~ 0.37 eV above the bottom of the conduction band minimum of *p*−Gr as one can see from Figure S1g. After the Li penetration is completed, the band gap of *p*−Gr/Li/Si reduced to ~ 0.02 eV, and the Dirac point largely recover and with the E_F passing though the Dirac point as shown in Figure S1h. The recovery of the Dirac band structure in the *p*-Gr can be attributed to the predominated bonding between Li and Si substrate in the Li intercalated structure (*p*−Gr/Li/Si).

DV5−8−5 defect exhibits a Dirac−like cone band structure but a ~ 0.30 eV gap opens between the Dirac−like cones as shown in Figure S2a. There is a band above the E_F level and within the gap between the Dirac−like cones. Figure S8 describes its PDOS of the typical C atoms around the defect region labeled in Figure S3, and shows that the main contribution to the narrow band in the gap is from the p_z states of C1 and C2 atoms near the defect. When Li atom adsorbs on the defect site of DV5−8−5 (i.e., Li/DV5−8−5), the band in the gap becomes more dispersive and partially occupied as showed in Figure S2b, which results in a p-type doping and enhanced conductivity. At the same time, the gap between the Dirac−like cones reduces to about 0.25 eV. For the saddle-point structure Li@DV5−8−5, the gap between the Dirac−like cones becomes wider (~ 0.39 eV) again as shown in Figure S2c. Meanwhile the dispersion of the band in the gap is slightly reduced but remains partial occupied. For Li/DV5−8−5 and Li@DV5−8−5, Figure S9 shows the PDOS of Li atom and the typical C atoms labeled in Figure S3. The p_z states of both

structures are mainly localized on the C1 atom, there are also slightly electron states on C2 and C3 atoms. Table S1 shows that Li atom maintains Li^{+} ion during Li diffusion. Similar to the case of *p*−Gr, the band structure recovered as that shown in Figure S2b after Li penetration is completed. When Si substrate is included, the DV5−8−5 still exhibits a Dirac−like cone structure. The gap between the cones becomes wider (~ 0.35 eV) comparing with the one of DV5−8−5/Si. There are three flat bands in the gap due to the influence Si substrate, and these bands are all above E_F as shown in Figure S2d. Upon Li adsorption, the bands in the gap become more complex and some of the bands become more dispersive and partially occupied as showed in Figure S2e. Similar to the case of Li/DV5−8−5, for Li/DV5−8−5/Si, Li adsorption leads to a p-doping and enhancement in conductivity. At the same time, the gap between the Dirac−like cones is significantly reduced to ~ 0.20 eV. For the saddle point structure Li@DV5−8−5/Si, the gap between the Dirac-like cone increases to ~ 0.39 eV, also similar to the case of Li@DV5−8−5 discussed above. After Li penetration is completed, the gap between the Dirac−like cones is ~ 0.33 eV, slightly smaller than the value in DV5−8−5/Si. Some of the bands are also partially occupied as shown in Figure S2g. During Li diffusion in DV5−8−5 and DV5−8−5/Si, the system remain p-type doping and Li atom maintains Li^{+} ion as shown in Table S1. The DV5−8−5 sheet gets all the valence electron of Li atom. In DV5−8−5/Li/Si, about 2/3 of the electron donated by the Li atom goes to the DV5−8−5 sheet and only 1/3 of electron donated by the Li atom transfers to the Si substrate. The charge transfer behavior indicates different bonding strength between Li and Si substrate and Gr sheet in the Li intercalated structure Gr/Li/Si.

Table S1. The bader charges of Li atom, Gr (Gr = *p*−Gr and DV5−8−5) sheet and Si substrate of the initial, saddle and end structures during Li diffusion in Gr and Gr/Si.

Structures	Li atom	Gr	Si Substrate
p−Gr		288.0000	
Li/*p*−Gr	0.0040	288.9960	
Li@*p*−Gr	0.0000	289.0000	
DV5−8−5		280.0000	
Li/DV5−8−5	0.0000	281.0000	
Li@DV5−8−5	0.0000	281.0000	
Si Substrate			272.0000
p−Gr/Si		287.9228	272.0772
Li/*p*−Gr/Si	0.0036	288.4992	272.4972
Li@*p*−Gr/Si	0.0000	288.5148	272.4852
p-Gr/Li/Si	0.0000	288.1979	272.8021
DV5−8−5/Si		280.0765	271.9235
Li/DV5−8−5/Si	0.0000	281.0790	271.9210
Li@DV5−8−5/Si	0.0000	281.0624	271.9376
DV5−8−5/Li/Si	0.0000	280.6576	272.3424

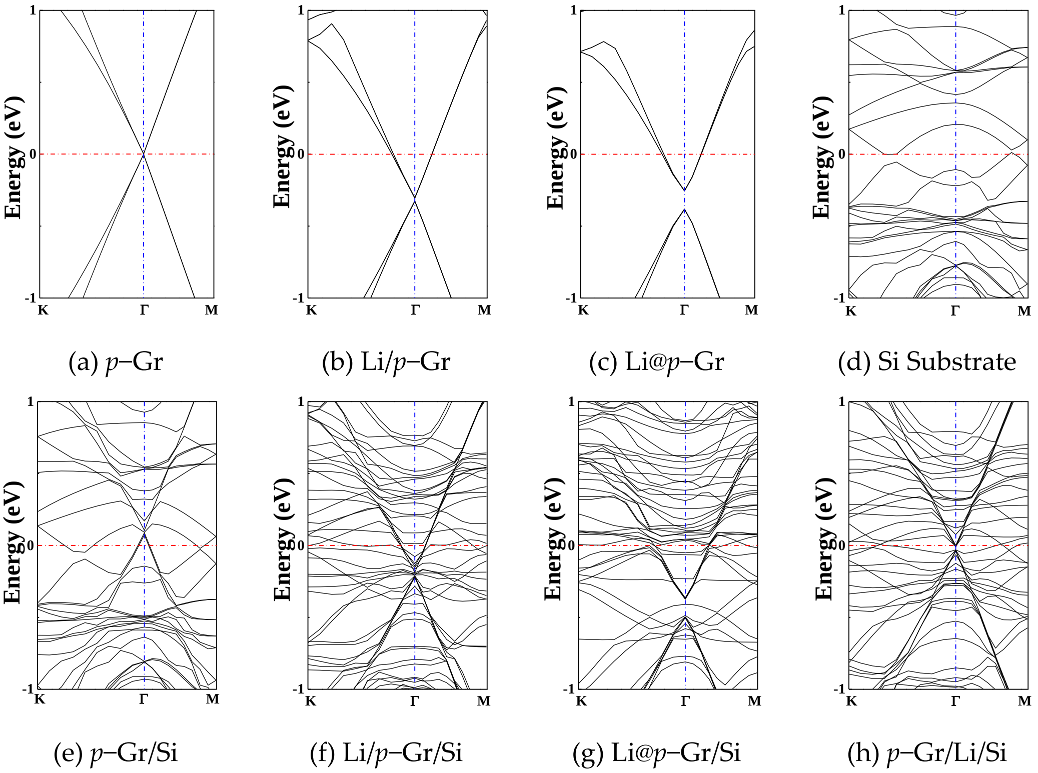

Figure S1. Band structures of (a) *p*−Gr (b) Li/*p*−Gr (c) Li@*p*−Gr (d) Si Substrate (e) *p*−Gr/Si (f) Li/*p*−Gr/Si (g) Li@*p*−Gr/Si (h) *p*−Gr/Li/Si.

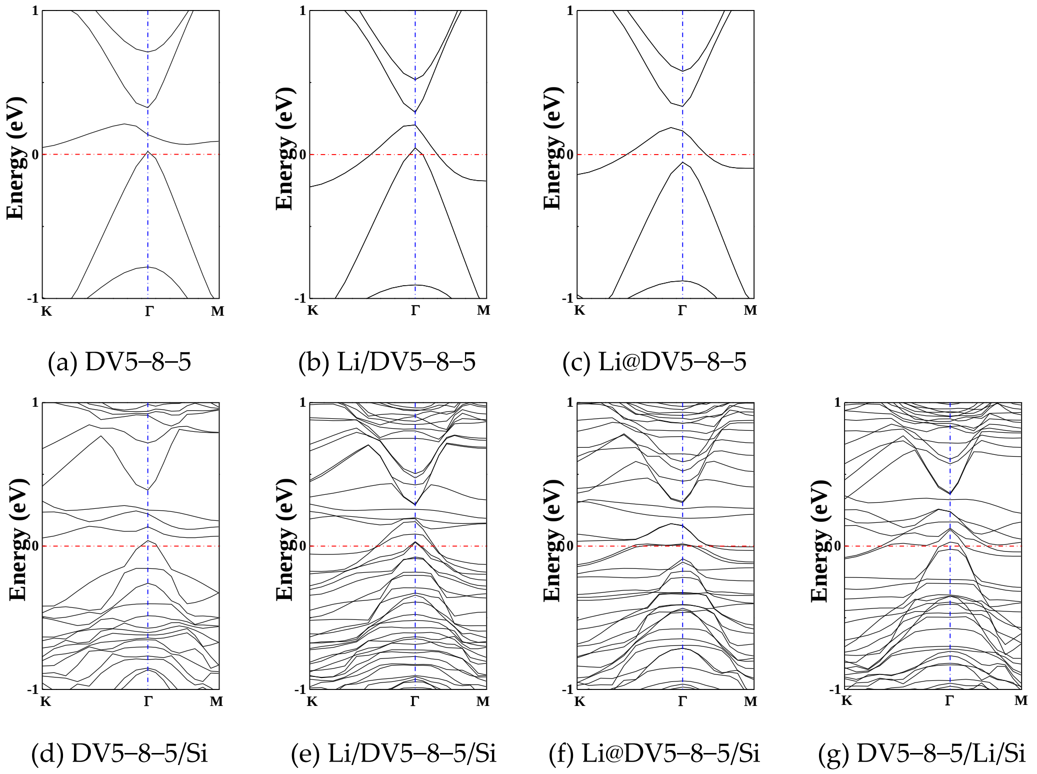

Figure S2. Band structures of (a) DV5−8−5 (b) Li/DV5−8−5 (c) Li@DV5−8−5 (d) DV5−8−5/Si (e) Li/DV5−8−5/Si (f) Li@DV5−8−5/Si and (g) Li/DV5−8−5/Si.

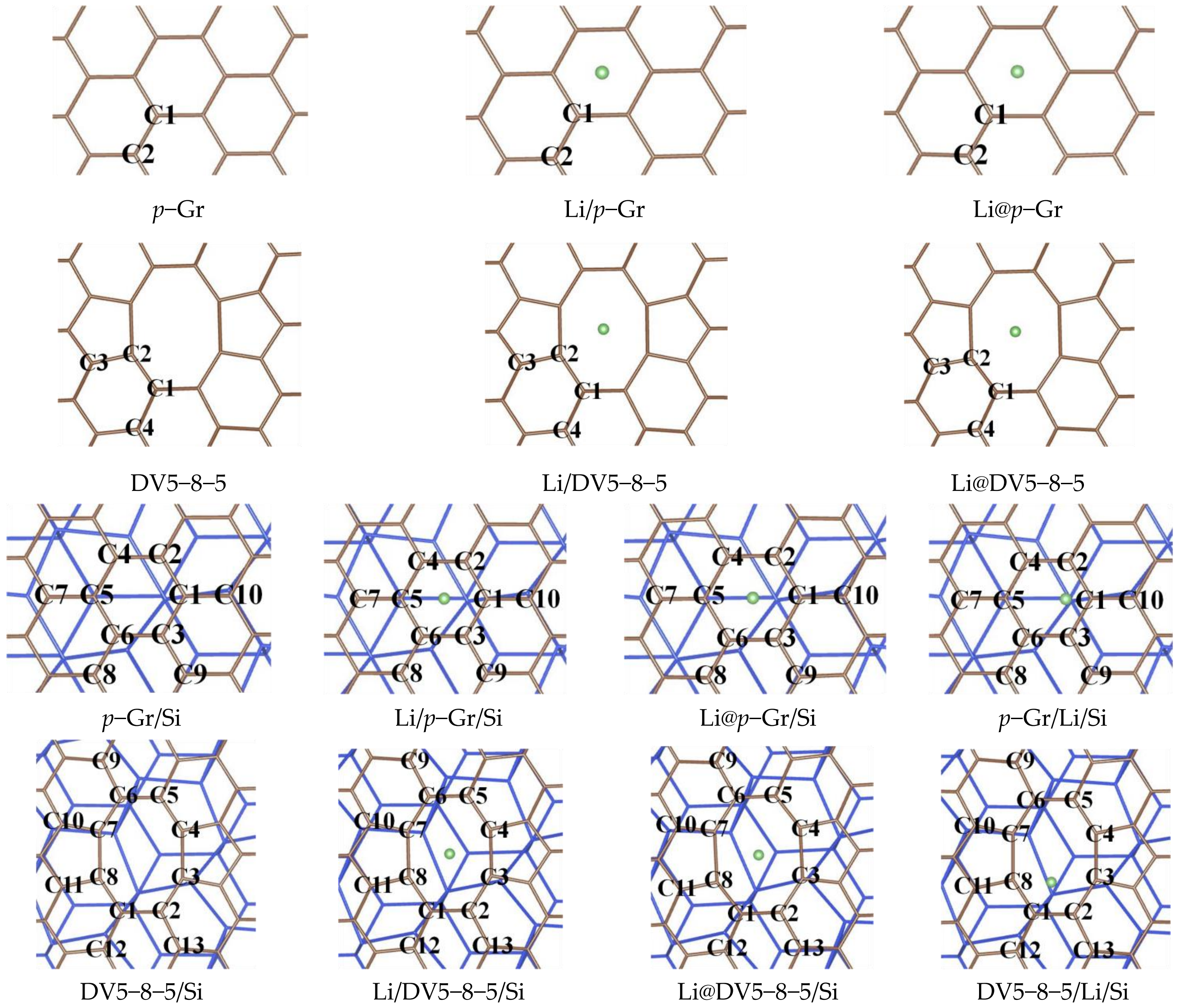

Figure S3. The line configurations of the initial, saddle and end structures during Li diffusion in Gr and Gr/Si (Gr = *p*–Gr and DV5–8–5); and the typical C atoms discussed in Figures S4 – S11 are labeled. The green ball represents Li atom; and the brown and blue lines represent C atoms and Si atoms and their bonding, respectively.

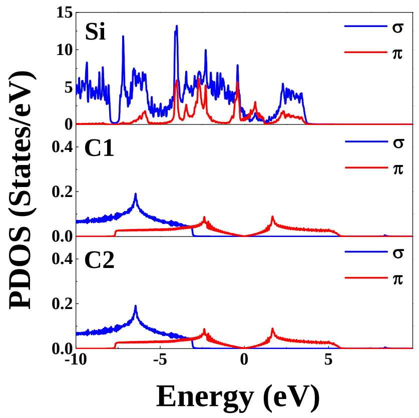

Figure S4. PDOS of the typical C atoms (labeled in Figure S3) of *p*–Gr and all Si atoms of Si substrate.

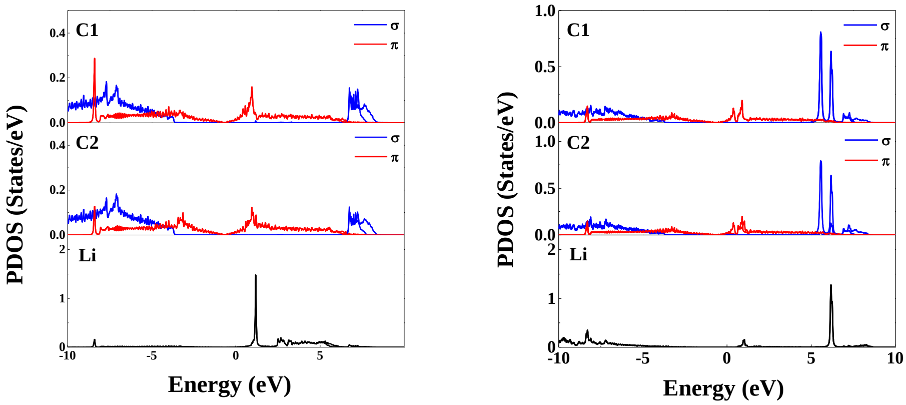

(a) Li/*p*–Gr (b) Li@*p*–Gr

Figure S5. PDOS of Li atom and the typical C atoms (labeled in Figure S3) in (a) Li/*p*–Gr and (b) Li@*p*–Gr.

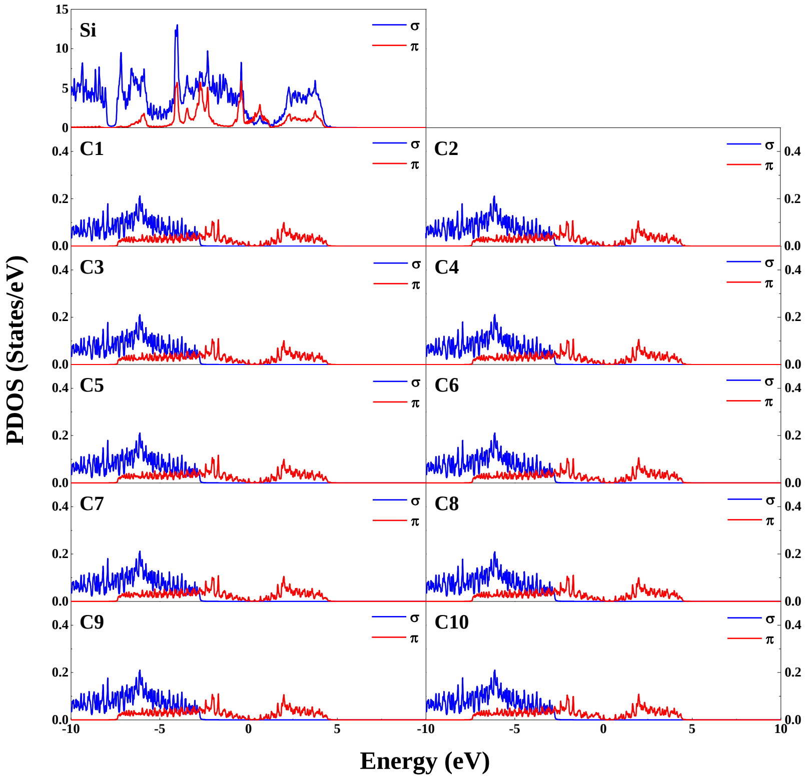

Figure S6. PDOS of the typical C atoms (labeled in Figure S3) and all Si atoms in *p*–Gr/Si.

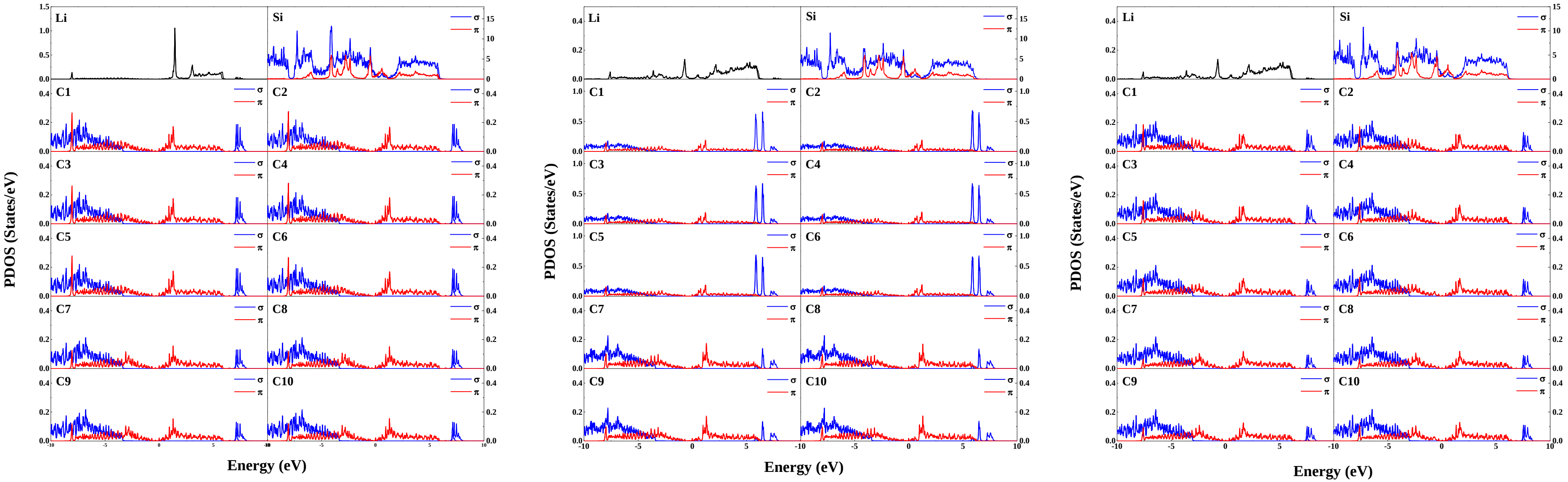

(a) Li/*p*−Gr/Si (b) Li@*p*−Gr/Si (c) *p*−Gr/Li/Si

Figure S7. PDOS of Li atom, all Si atoms and the typical C atoms (labeled in Figure S3) in (a) Li/*p*−Gr/Si (b) Li@*p*−Gr/Si and (c) *p*−Gr/Li/Si.

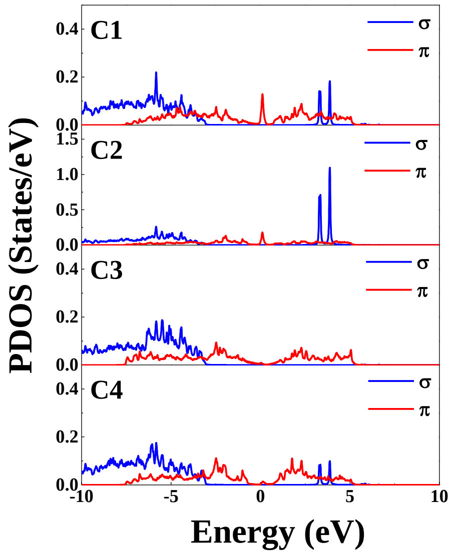

Figure S8. PDOS of the typical C atoms in DV5–8–5 labeled in Figure S3.

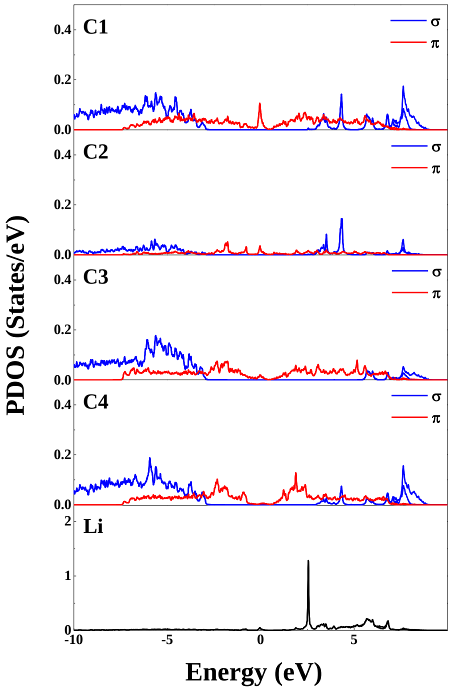

(a) Li/DV5–8–5

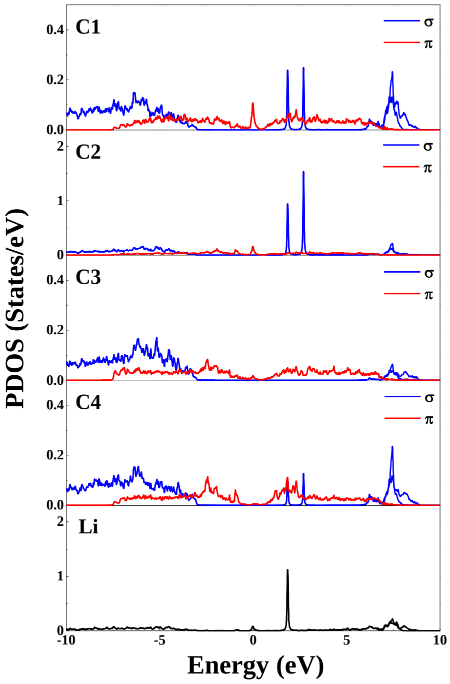

(b) Li@DV5–8–5

Figure S9. PDOS of Li atom and the typical C atoms (labeled in Figure S3) in (a) Li/DV5–8–5 and (b) Li@DV5–8–5.

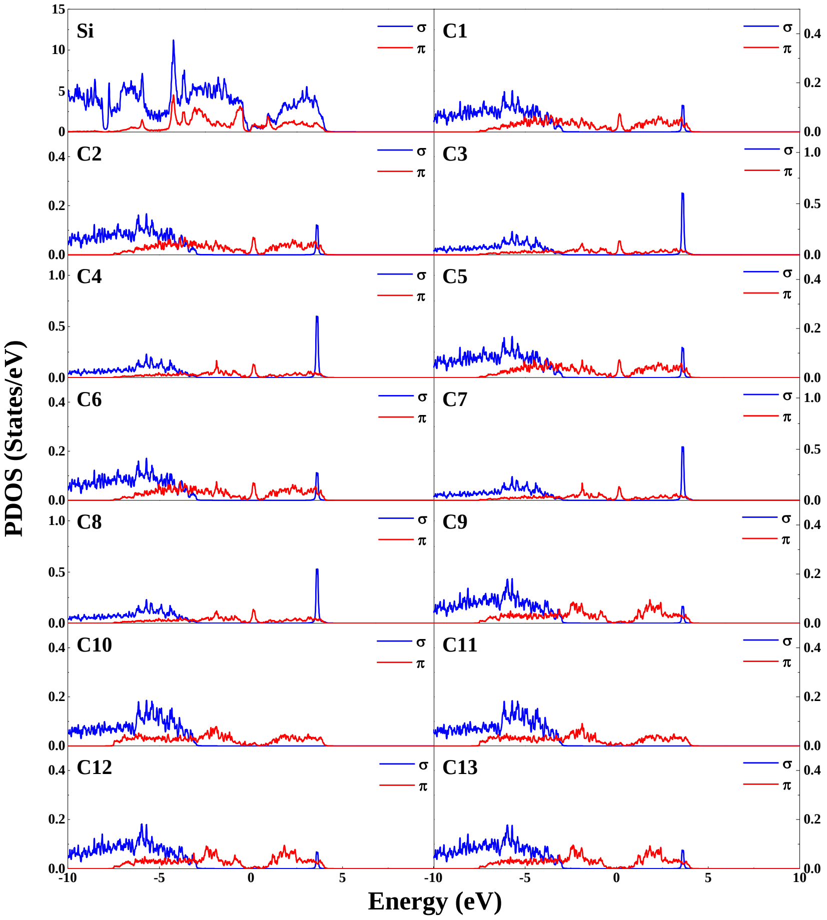

Figure S10. PDOS of all Si atoms of the typical C atoms (labeled in Figure S3) in DV5–8–5/Si.

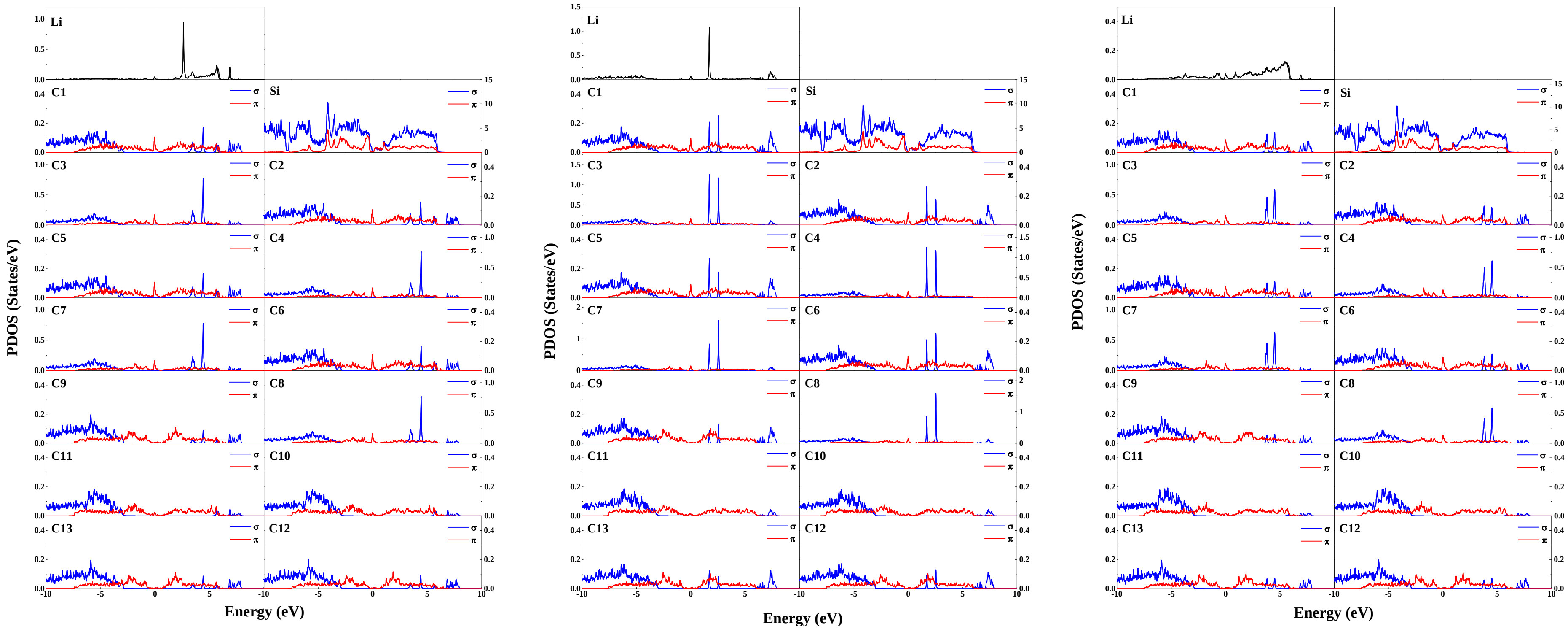

(a) Li/DV5–8–5/Si (b) Li@DV5–8–5/Si (c) DV5–8–5/Li/Si

Figure S11. PDOS of Li atom, all Si atoms and the typical C atoms (labeled in Figure S3) in (a) Li/DV5–8–5/Si (b) Li@DV5–8–5/Si and (c) DV5–8–5/Li/Si.
